# SAAMBE-MEM: a sequence-based method for predicting binding free energy change upon mutation in membrane protein–protein complexes

**DOI:** 10.1093/bioinformatics/btae544

**Published:** 2024-09-06

**Authors:** Prawin Rimal, Shailesh Kumar Panday, Wang Xu, Yunhui Peng, Emil Alexov

**Affiliations:** Department of Physics and Astronomy, Clemson University, Clemson, SC 29634, United States; Department of Physics and Astronomy, Clemson University, Clemson, SC 29634, United States; Institute of Biophysics and Department of Physics, Central China Normal University, Wuhan, Hubei 430079, China; Institute of Biophysics and Department of Physics, Central China Normal University, Wuhan, Hubei 430079, China; Department of Physics and Astronomy, Clemson University, Clemson, SC 29634, United States

## Abstract

**Motivation:**

Mutations in protein–protein interactions can affect the corresponding complexes, impacting function and potentially leading to disease. Given the abundance of membrane proteins, it is crucial to assess the impact of mutations on the binding affinity of these proteins. Although several methods exist to predict the binding free energy change due to mutations in protein–protein complexes, most require structural information of the protein complex and are primarily trained on the SKEMPI database, which is composed mainly of soluble proteins.

**Results:**

A novel sequence-based method (SAAMBE-MEM) for predicting binding free energy changes (ΔΔ*G*) in membrane protein–protein complexes due to mutations has been developed. This method utilized the MPAD database, which contains binding affinities for wild-type and mutant membrane protein complexes. A machine learning model was developed to predict ΔΔ*G* by leveraging features such as amino acid indices and position-specific scoring matrices (PSSM). Through extensive dataset curation and feature extraction, SAAMBE-MEM was trained and validated using the XGBoost regression algorithm. The optimal feature set, including PSSM-related features, achieved a Pearson correlation coefficient of 0.64, outperforming existing methods trained on the SKEMPI database. Furthermore, it was demonstrated that SAAMBE-MEM performs much better when utilizing evolution-based features in contrast to physicochemical features.

**Availability and implementation:**

The method is accessible via a web server and standalone code at http://compbio.clemson.edu/SAAMBE-MEM/. The cleaned MPAD database is available at the website.

## 1 Introduction

The protein–protein interactions (PPIs) are fundamental for numerous vital biological processes, such as cell regulation, metabolism, transport (Paumi *et al.* 2007), and signaling (Chuderland and Seger 2005), and are intricately affected by amino acid mutations (Petukh *et al.* 2015). These mutations can either be detrimental, leading to diseases (David *et al.* 2012, Gao *et al.* 2015) by altering normal functions, or beneficial, contributing to genetic diversity and adaptation (Lande and Shannon 1996). Assessing the effects of mutations on PPIs requires a detailed analysis of how they modify the proteins’ interaction dynamics, including changes in binding affinities. Such evaluations are crucial for advancing protein engineering by designing proteins with high affinity (Grönwall and Ståhl 2009) for their partners, developing novel therapeutic strategies (Wells and McClendon 2007), and understanding molecular mechanisms of disease (Kuzmanov and Emili 2013). Assessing these effects requires joint efforts of both experimental (Fragoza *et al.* 2019) and computational (Das *et al.* 2012) approaches.

PPIs occur in various conditions and cellular environments; however, they can be broadly grouped into two categories: PPIs of soluble and PPIs of membrane proteins. Soluble proteins carry out their function in an aqueous environment, and thus, their surface is enriched by hydrophilic residues, including the binding interface. Experimental data about the binding free energy of wild type and mutant proteins was collected in various databases, the most prominent being the SKEMPI database (Jankauskaitė *et al.* 2019). On the other side of the spectrum are PPIs involving membrane proteins. Approximately 20%–30% (Krogh *et al.* 2001) of all proteins in living organisms are membrane proteins, highlighting their essential role in cellular function. Membrane proteins contain a higher proportion of hydrophobic amino acids, enabling their integration into the hydrophobic lipid bilayer. Lipids, with their hydrophobic tails and hydrophilic heads, form a bilayer that creates a distinct environment for membrane proteins, quite different from the environment of soluble proteins. The available experimental data of binding free energy of wild type and mutant membrane proteins was recently collected into a database, the MPAD database (Ridha *et al.* 2023). The existence of two databases, the SKEMPI and MPAD databases, along with the different environments of the corresponding PPIs mentioned above, suggests that methods and approaches designed to predict binding free energy changes caused by mutations for soluble proteins may not be suitable for membrane PPIs. Indeed, as it will be demonstrated in the results section, the methods trained on databases containing soluble PPIs do not perform well on membrane PPIs. This motivates us to develop a method that predicts the changes of the binding free energy caused by mutations for membrane proteins.

Another distinction between soluble and membrane protein–protein complexes is the availability of 3D structures. While there are many structures of soluble protein–protein complexes deposited in protein data bank (PDB) (Berman *et al.* 2002), just a tiny fraction of membrane proteins and their complexes is available. The availability of 3D structures of soluble protein–protein complexes resulted in the fact that the vast majority of the methods for predicting the change of the binding free energy caused by mutation for soluble proteins (Dehouck *et al.* 2013, Xiong *et al.* 2017, Geng *et al.* 2019, Rodrigues *et al.* 2019, Jemimah *et al.* 2020, Pahari *et al.* 2020, Li *et al.* 2021) are structure-based, i.e. they require 3D structure of the protein–protein complex in order to make the predictions (list of the most popular methods is provided in Pandey *et al.* 2023). However, structure-based approaches are not efficient for modeling membrane PPIs since very few 3D structures are available. This motivates us to develop a method that is sequence-based and does not require structural information.

It should be clarified that while the MPAD database is referred to as a database of experimental data on the binding free energy of wild-type and mutant membrane proteins, the vast majority of the entries are protein domains that are not embedded in the membrane. These domains are either peripheral membrane proteins or domains linked to transmembrane proteins but do not reside within the lipid bilayer. In other words, most of the entries in MPAD are proteins or domains that are fully or partially solvated in the water phase. Despite this, existing methods for predicting ΔΔ*G* of PPIs do not perform well on MPAD cases, and the distribution of ΔΔ*G* in MPAD is quite different from that in SKEMPI (as will be shown in the results section). This indicates that the presence of the membrane and the lipid bilayer provides a unique environment that perturbs the bulk water properties and results in PPIs that differ from those in bulk water.

## 2 Materials and methods

### 2.1 Dataset creation (cleaning MPAD database)

The original MPAD (Ridha *et al.* 2023) database is composed of binding affinities of wild-type and mutant membrane protein–protein complexes upon single, double, and multiple mutations collected from several experimentally reported sources. From the binding affinity *K_D_*, binding free energy Δ*G* for both the wild-type and mutant complexes can be computed as:

(1) $$\Delta G=RT\;\text{ln}\left(K_{D}\right),$$

where *R* is ideal gas constant and *T* is the absolute temperature. The change in binding free energy, ΔΔ*G*, is defined as the difference in binding free energy between the mutant and wild-type complexes and is given as:

(2) $$\Delta \Delta G=\Delta G_{\text{mutant}}-\Delta G_{\text{wild}-\text{type}.}$$

and thus, a positive ΔΔ*G* indicates that the mutation destabilizes the complex, while negative ΔΔ*G* indicates the opposite.

For our purpose, we are concerned with binding affinities of dimeric complexes with single mutations. The database contains 2792 single mutations corresponding to 264 distinct proteins. Initially, discrepancies were noted between the provided ΔΔ*G* values and the difference between mutated Δ*G*_mutant_ and wild-type Δ*G*_wild-type_ values, prompting necessary corrections. In addition, it was observed that not all complexes had associated PDB IDs or ΔΔ*G* values. To address this issue, pseudonyms were assigned to each complex with missing PDB IDs for clear identification. Excluding the mutations lacking ΔΔ*G* values yielded a dataset of 2679 mutations and 246 proteins. For instances where proteins shared identical mutations, the mean and standard deviation of ΔΔ*G* were computed. Mutations with a standard deviation >1.0 kcal/mol were subsequently eliminated, resulting in a refined dataset of 2647 mutations and 246 proteins.

Furthermore, unique instances of identical mutations with ΔΔ*G* values opposite in sign or magnitude were addressed following a predetermined rule, leading to a dataset of 2618 mutations and 245 proteins. In situations where two cases displayed opposite signs and ΔΔ*G* values exceeding an absolute threshold of 0.5 kcal/mol, both instances were removed. Similarly, cases with three or four instances featuring different signs and any ΔΔ*G* values surpassing an absolute threshold of 0.5 kcal/mol were eliminated. In addition, for cases involving multiple instances where only one or two ΔΔ*G* values deviated significantly, they were selectively removed, and a recalculated mean was derived from the remaining cases. After refining, duplicate mutations were merged into a single representation, using the mean ΔΔ*G* value, yielding a curated dataset of 2148 cases and 245 proteins.

The cases missing a UniProt (UniProt Consortium 2019) ID for at least one of the binding proteins, along with the missing corresponding PDB (Berman *et al.* 2002) structure of the complex, were deleted, resulting in 1888 cases and 204 proteins. Mutations not adhering to the standard format of residue name and position were discarded, leading to 1863 cases and 196 proteins. For training with a sequence-based approach, the stated residue in the database had to be present in the UniProt sequence or the sequence imported from the PDB structure in case of a missing UniProt (UniProt Consortium 2019) ID. The structure residue number in the MPAD database was translated to the sequence residue number for all cases. Cases not meeting this criterion were further removed, leading to 1579 cases and 143 proteins. Finally, only cases of dimeric membrane protein were retained, resulting in a final 1040 mutations from 89 proteins and the database is termed MPAD_Clean.

### 2.2 Features used in the machine learning algorithm

#### 2.2.1 Amino acids index (AAIndex)

The compilation consists of experimental and computed values across three sections, representing a broad spectrum of physicochemical and biochemical characteristics of amino acids and amino acid pairs (Kawashima *et al.* 2007), and is available at https://www.genome.jp/dbget/aaindex.html. Amino Acid Index 1 (AAIndex1) encompasses the physicochemical attributes of amino acids and contains 566 indices. After eliminating cases of missing values, 547 indices were used as predictors by calculating the difference between the attribute values of the mutant and the wildtype amino acids. Meanwhile, Amino Acid Index 2 (AAIndex2) comprises 94 mutation matrices for pairs of amino acids, providing the scores for substituting one amino acid with another. Amino Index 3 (AAIndex3) includes the statistical contact potential, which requires structural information of proteins and is not utilized as a training feature.

#### 2.2.2 Position-specific scoring matrix

A Position-Specific Scoring Matrix (PSSM) is a matrix that indicates the likelihood of the occurrence of an amino acid at a specific position within an aligned set of sequences. The scores are generated based on the observed frequencies of amino acids in the aligned sequences, considering the background probabilities and evolutionary relationships. For our analysis, we generated the PSSM by performing PSI-BLAST (Camacho *et al.* 2009) against the UniRef50 (Suzek *et al.* 2015) database over three iterations, with a cut-off E-value of 0.001. The resulting matrix is an L × 20 matrix, where L is the length of the sequence with as many rows and each of the 20 columns representing one of the amino acids.

(3) $$P_{\text{PSSM}}=\left(\begin{array}{cccc} P_{1,1} & P_{1,2} & \cdots & P_{1,20}\\ P_{2,1} & P_{2,2} & \cdots & P_{2,20}\\ \vdots & \vdots & \vdots & \vdots \\ P_{L,1} & P_{L,2} & \cdots & P_{L,20} \end{array}\right),$$

where *P_i,j_* gives the log-score for amino acid in the *i*th position of protein mutating to *j*th amino acid.

The matrix elements are then normalized between 0 and 1 using a sigmoid function as

(4) $$f(x)=1/\left(1+e^{-x}\right),$$

where *x* is the element of PSSM matrix given in the expression (3).

Subsequently, the matrix is averaged across columns for all 20 amino acids. The averaging makes the matrix independent of sequence length, which is a necessary step to ensure uniformity when using it as a training feature for all proteins as

(5) $${\bar{P}}_{j}=\frac{1}{L}\sum_{i=1}^{L}P_{i,j}\;\left(j=1,\;2,\;3,\ldots .,20\right).$$

The set of 20 numbers, obtained after averaging and normalization, is generated for both the interacting and mutating sequences. This set is referred to as the PSSM hereafter.

(6) $${\bar{P}}_{\text{PSSM}}={\left({\bar{P}}_{1},\;{\bar{P}}_{2},\ldots \ldots ,{\bar{P}}_{20}\right)}^{T}.$$

In addition to the normalized average PSSM of the overall sequence, the row scores corresponding to the mutating amino acid site from the PSSM matrix are also used as a feature to capture the evolutionary properties of the mutating site. This set of scores is referred to as the Row-PSSM.

#### 2.2.3 Pseudo position-specific scoring matrix (Pse-PSSM)

While the PSSM and Row-PSSM capture the overall sequence properties and the site-specific properties of the amino acids, respectively, the pseudo position-specific scoring matrix (Shen and Chou 2007) is defined to capture the sequence order properties of the amino acids as follows:

(7) $$\phi_{j}^{\varphi }=\frac{1}{L-\varphi }\sum_{i=1}^{L-\varphi }{\left(P_{i,j}-P_{\left(i+\varphi \right),j}\right)}^{2}\;\left(j=1,2,3,\ldots \ldots ,20;0<\varphi <L\right).$$

It is generated for both the interacting and mutating protein sequences with φ = 1 to 10 as:

(8) $$P_{\text{PsePSSM}}={\left({\bar{P}}_{1},\;{\bar{P}}_{2},\ldots ,{\bar{P}}_{20};\phi_{1}^{1},\;\phi_{2}^{1},\ldots \ldots ,\phi_{20}^{1};\ldots \ldots \ldots ;\phi_{1}^{\varphi },\;\phi_{2}^{\varphi }\ldots \ldots \phi_{20}^{\varphi }\right)}^{T}.$$

#### 2.2.4 Database features

Features such as the types of membrane proteins, in terms of their function and structure, and experimental conditions like pH values, which cannot be derived from sequence information but are included in this dataset, are referred to as Database features and are used for training.

#### 2.2.5 Neighboring amino acid index

The leading and lagging five amino acids from the mutating sites are indexed and used as features, referred to as NeighborAA hereafter.

#### 2.2.6 Mutation type

Each possible mutation pair, formed from the wild-type and mutated amino acids, is labeled by a pair of numbers. With 20 amino acids, there are 380 possible mutation types and an equal number of labels.

#### 2.2.7 Amino acid category features

This includes features based on the categories of wildtype and mutated amino acids. The classification is made based on the properties of the side chain of the amino acids. These categories result in various labels representing changes between different properties caused by mutation. The features are indexed for training purposes, abbreviated as CategoryAA hereafter, and includes the following:

1. Chemical property
  Amino acids are classified into seven categories based on the chemical properties: basic (H, K, R), amide (N, Q), acidic (D, E), sulfur (C, M), hydroxyl (S, T), aromatic (F, W, Y), and aliphatic (A, G, I, L, P, V).
2. Size
  Amino acids are categorized into five classes based on the size: small (C, D, N, P, T), medium (E, H, Q, V), large (I, K, L, M, R), very large (F, W, Y), and very small (A, G, S).
3. Polarity
  Polarity is divided into four types: polar basic (H, K, R), polar neutral (N, Q, S, T, Y), polar acidic (D, E), and nonpolar (A, C, F, G, I, L, M, P, V, W). Different labels represent changes caused by mutations between polar to polar, polar to nonpolar, nonpolar to polar, nonpolar to nonpolar and so on.
4. Hydrogen bonding
  Hydrogen bonding is classified into four categories: donor (K, R, W), acceptor (D, E), donor and acceptor (H, N, Q, S, T, Y), and none (A, C, F, G, I, L, M, P, V).
5. Hydrophobicity
  Hydrophobicity is divided into three classes: hydrophobic (A, C, F, I, L, M, V, W), neutral (G, H, P, S, T, Y), and hydrophilic (D, E, K, R, N, Q).

#### 2.2.8 Features categorization

All the features used for training described earlier can be broadly classified into three categories: physicochemical, evolutionary, and database features. The physicochemical and evolutionary features can be obtained from the sequence information of the involved proteins, but the database features cannot be.

**Figure 1.**
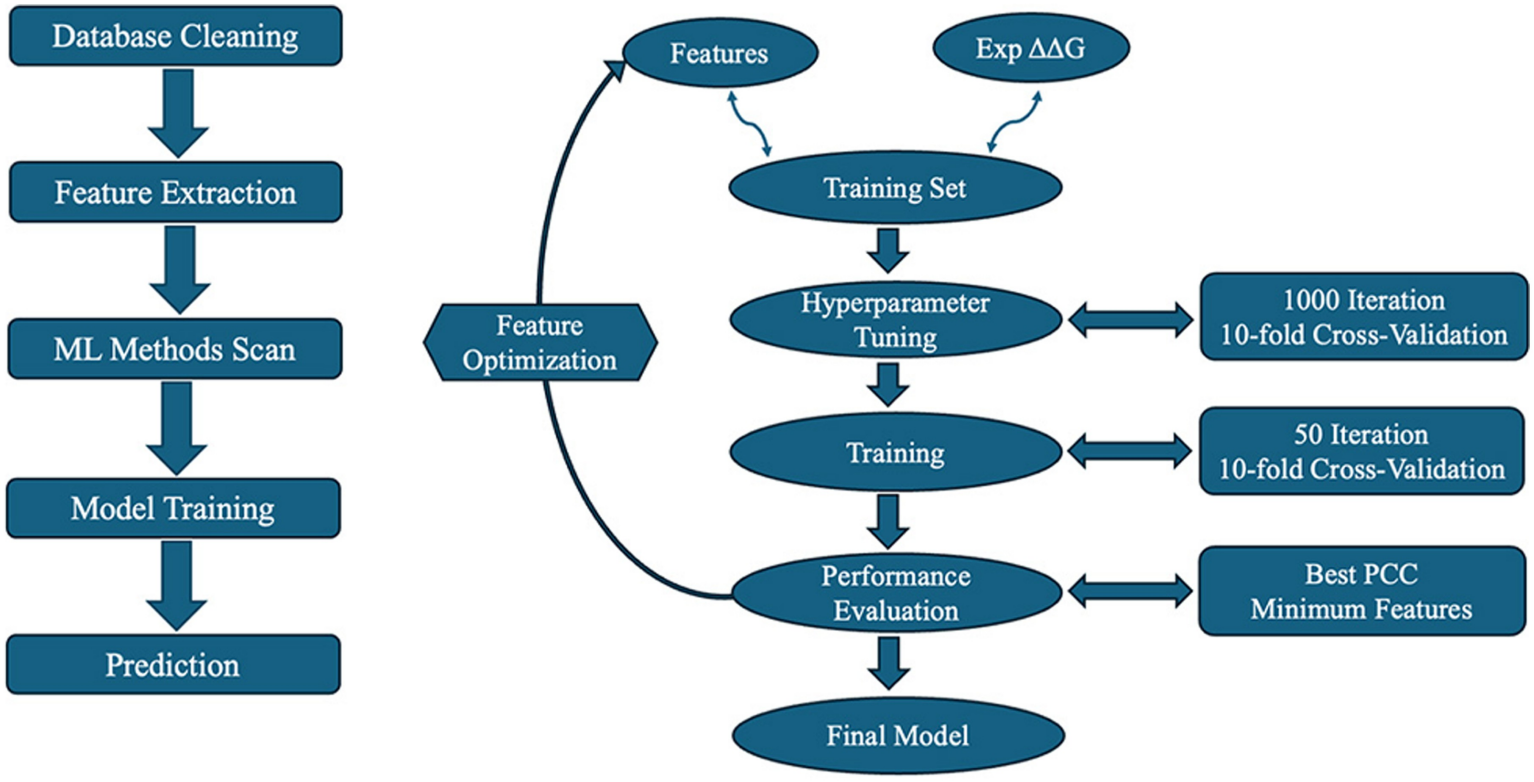
The flowchart representation of machine learning.

1. Physicochemical features, abbreviated as “Phychem” features, are directly measured or represented from the physical and chemical properties of amino acids. These include AAIndex 1, the features listed under Amino Acid Category Features, and the Neighboring Amino Acid Index.
2. Evolutionary features document the statistical occurrence and substitution of amino acids in proteins. These include AAIndex 2, PSSM, Row-PSSM, and Pse-PSSM.
3. Database features are obtained from the database and cannot be generated solely from sequence information. They include characteristics such as the types of membrane proteins in terms of their function and structure, and experimental conditions like pH values (Fig. 1).

### 2.3 Model training with cross-validation

To avoid overfitting or underfitting of the model, it is crucial to perform cross-validation and hyperparameter tuning during the learning and testing phases. We used 5-fold as well as 10-fold cross validation. In *k*-fold cross-validation, the dataset is randomly divided into *k*-equal parts, (*k*-1) parts are used to train the model, and the remaining 1 part/fold is used to test the model's performance. This process was repeated *k* times, with each of the *k*-folds being used as the testing fold in subsequent iterations.

Hyperparameters are a set of criteria that are set prior to the initiation of the learning process and are used to control the training process. Hyperparameter tuning was conducted through 1000 iterations of 5-fold/10-fold cross-validations on the training dataset by randomly selecting hyperparameters from a predefined dictionary listed in Supplementary Table S1. The optimal set of hyperparameters was determined as the one that yielded the highest correlation between the experimental and predicted ΔΔ*G* values for that training set.

Upon determining the best set of hyperparameters for a training set, 50 iterations of 5-fold/10-fold cross-validations were performed. This process was repeated with several subsets of features to achieve the best performance with the minimum or optimal number of features. In addition, the performances of training sets with evolutionary and physicochemical features were also compared. After comparing performance of 5-fold and 10-fold cross validations, the outcomes were found to be similar (see section 3.3). Thus, we choose to present detailed discussion for models using 10-fold cross validation only (Fig. 1).

### 2.4 Choosing a machine learning method

The set of all the features extracted from the sequence of binding proteins and experimental ΔΔ*G* values forms the entire training set. Predicting the binding free energy change (ΔΔ*G*), a continuous value, requires a machine learning method based on a regression model. For this purpose, the AutoML tool from the PyCaret library was utilized for initial and quick training to evaluate the performance of various regression models on our training dataset with all the features. The top seven regression models and their respective performance during training with PyCaret are presented in the Table 1.

**Table 1.** Performance of top seven regression models from PyCaret on MPAD database.

Regressor models	PCC	RMSE (kcal/mol)
Extra Trees	0.54	0.88
Random Forrest	0.52	0.88
Light Gradient Boosting	0.47	0.92
Gradient Boosting	0.47	0.92
Extreme Gradient Boosting	0.44	0.96
AdaBoost	0.43	0.93
Decision Tree	0.30	1.18

In the AutoML library, hyperparameter tuning is conducted over a predefined small hyperparameter space which might not lead to the optimal performance. Hence, the top five performing regression models identified by AutoML, based on the correlation between the actual and predicted ΔΔ*G* values, were selected for further training with the dataset, utilizing all the features and subsequent extensive hyperparameter tuning. In this stage, a much larger hyperparameter space is scanned with 1000 iterations of training. Considering both time efficiency and performance in terms of PCC, Extreme Gradient Boosting: XGBoost (Chen and Guestrin 2016)—a tree-based regression machine learning model—was identified as the optimal choice for further analysis. The outcomes for all five methodologies are presented in Table 2.

**Table 2.** Performance of training top five regression models with the respective regressors with extensive hyperparameter tuning for the best iteration.

ML method	PCC	RMSE (kcal/mol)
Random Forrest	0.55	0.86
Extra Trees	0.55	0.86
Gradient Boosting	0.55	0.85
Light Gradient Boosting	0.57	0.85
Extreme Gradient Boosting	0.58	0.83

### 2.5 Feature importance analysis

**Figure 2.**
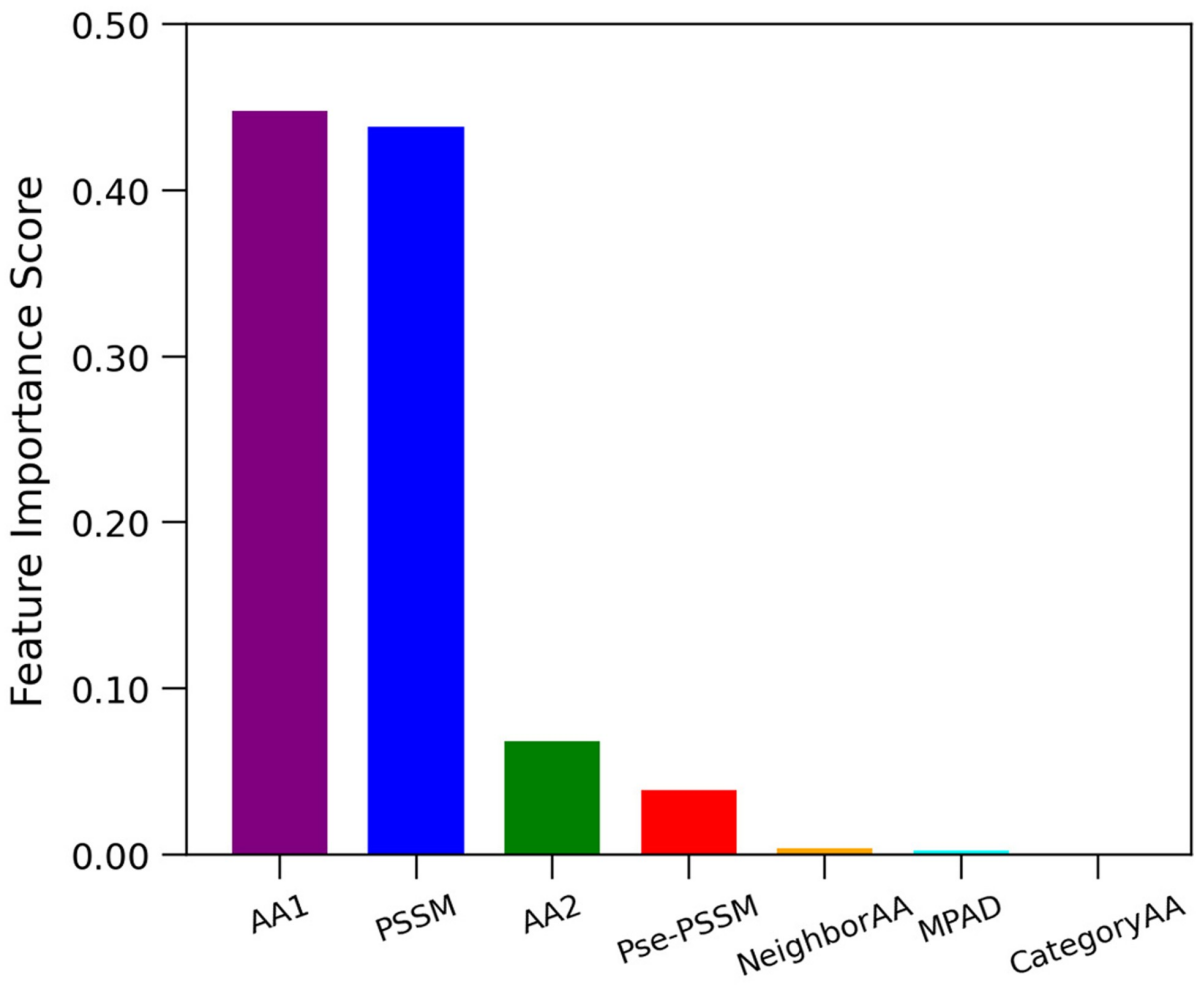
Cumulative feature importance scores of several categories of features.

The feature classes were ranked based on the average feature importance score, derived from conducting 100 iterations of 10-fold cross-validation while training the model with XGBoost. This methodology ensures a comprehensive assessment of feature relevance, facilitating the identification and prioritization of the most impactful variables in the predictive model’s performance (Fig. 2) (Table 3).

**Table 3.** Cumulative feature importance scores of several categories of features.

Feature	Importance score
AAIndex (AA1)	0.44743
PSSM	0.43810
AAIndex 2 (AA2)	0.06852
Pse-PSSM	0.03903
NeighborAA	0.00371
Database	0.00244
CategoryAA	0.00078

## 3 Results

### 3.1 Comparison of SKEMPI-V2 and MPAD database

The existing machine learning methods for predicting ΔΔ*G* due to mutations in protein–protein complexes were trained on the SKEMPI (Jankauskaitė *et al.* 2019) database, which is composed of various kinds of protein complexes. The MPAD (Ridha *et al.* 2023) database, on the other hand, consists entirely of membrane protein–protein complexes. Here, we analyze the similarities and differences between the SKEMPI-V2 and MPAD databases in terms of the protein complexes, associated mutations, and ΔΔ*G* values.

The SKEMPI-V2 and MPAD databases, curated for training with a sequence-based approach, contain 209 protein complexes with 2472 mutations and 89 membrane protein complexes with 1040 mutations, respectively. After comparison, an overlap between the protein complexes and mutation cases among the two databases was observed. Notably, 42.69% (38 out of 89) of the protein complexes present in the MPAD database matched those in the SKEMPI-V2 database. In addition, 564 mutations, representing 54.23% of the total cases present in the MPAD database, were also found in the SKEMPI-V2 database (Table 4). However, SKEMPI is a much larger database, and the inclusion of 564 mutations of membrane proteins constitutes only 22.80% of 2472 mutation cases in the SKEMPI database. This also implies that most of the mutations in the SKEMPI-V2 database are from soluble proteins, making a strong case for developing a new model trained on a database composed exclusively of membrane protein complexes (Table 4).

**Table 4.** Summary of protein complexes and mutations of SKEMPI-V2 and MPAD databases.

	Protein complex	Mutation cases
SKEMPI-V2	209	2472
MPAD	89	1040
Overlap Count	38	564
Overlap % (of MPAD)	42.69	54.23

**Figure 3.**
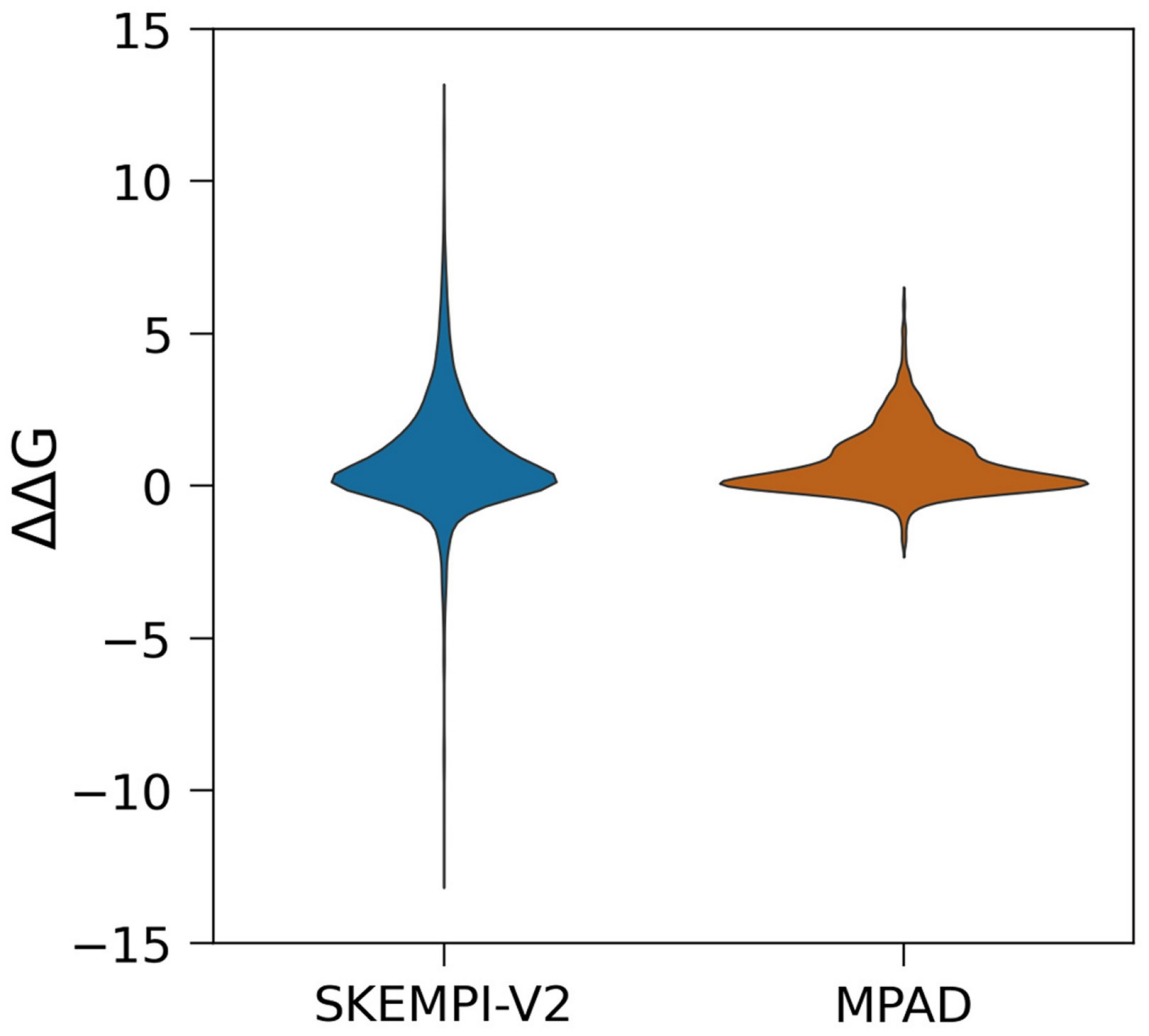
The distribution of ΔΔ*G* values of SKEMPI-V2 and MPAD databases in a violin plot.

The next question that was asked is to compare the corresponding ΔΔ*G* in SKEMPI and MPAD (Fig. 3 and Supplementary Fig. S1). One can see that the ΔΔ*G* values in both databases are mostly clustered around 0, indicating that the mutations generally do not significantly impact the binding free energy. However, this clustering is more pronounced in the MPAD database compared to the SKEMPI-V2 database.

**Figure 4.**
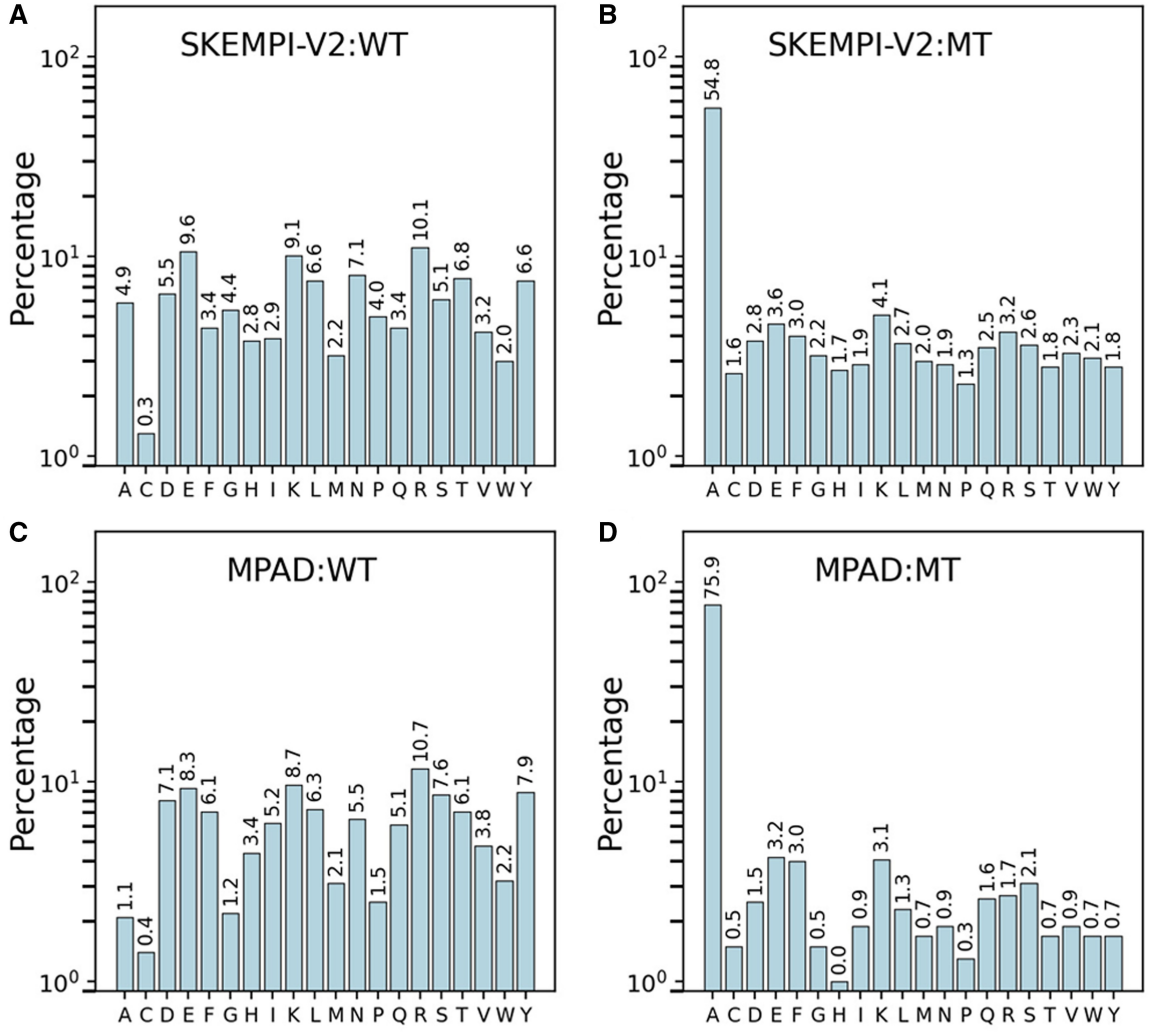
Comparison of distribution for wild-type and mutated amino acids for (top: A, B) SKEMPI-V2 and (bottom: C, D) MPAD databases.

In terms of the distribution of wild-type and mutated amino acids in the databases (Fig. 4 and Supplementary Table S2), it is observed that all amino acids are fairly represented in the wild-type sequences of both databases. However, alanine is highly overrepresented among the mutated amino acids due to the prevalence of alanine scanning in experiments. The percentages of alanine among the mutated amino acids are 54.8% and 75.9% for SKEMPI-V2 and MPAD, respectively. Apart from the difference between SKEMPI-V2 and MPAD in terms of the prevalence of alanine mutations, there is no significant difference in the mutation pattern. This indicates that the difference in ΔΔ*G* distribution and the inability of existing methods to correctly predict ΔΔ*G*s is due to other factors, namely the presence of the membrane.

### 3.2 Accessing the performance of SAAMBE-SEQ on MPAD database

**Figure 5.**
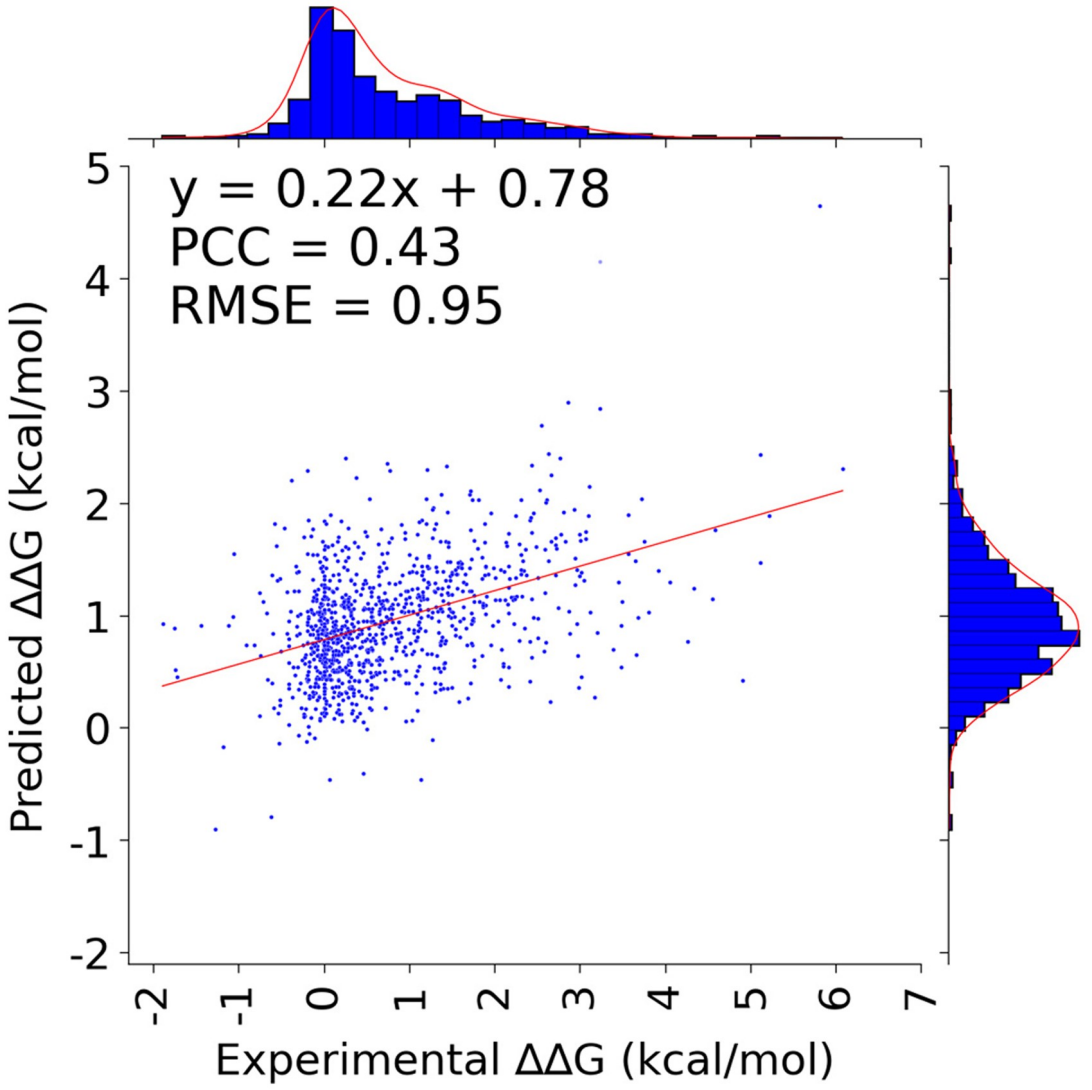
Performance of SAAMBE-SEQ on MPAD database.

SAAMBE-SEQ is the only operational method that uses sequence information and does not require the 3D structure of the corresponding protein–protein complex for prediction purposes [we tried ProAffiMuSeq (Jemimah *et al.* 2020), but it was not operational]. SAAMBE-SEQ was trained on the SKEMPI database, and one could ask if the method performs well on MPAD as well. This motivated us to evaluate the performance of SAAMBE-SEQ on the MPAD database. A Pearson correlation coefficient (PCC) of 0.43 was obtained with SAAMBE-SEQ on the MPAD database (Fig. 5), which is much less accurate than the PCC of 0.91 (10-fold cross-validation) obtained on the SKEMPI database. Furthermore, we investigated the performance of several structure-based methods (SAAMBE-3D and MutaBind2) on the MPAD entries with existing 3D structures that were not present in the SKEMPI database. The results are shown in Supplementary Fig. S2, and the corresponding PCCs are <0.3. This indicates that a new method should be developed.

### 3.3 Performance of the SAAMBE-MEM trained on MPAD database

We benchmarked SAAMBE-MEM on the MPAD-Clean database using different sets of features. The goal was 2-fold: to minimize the number of features while achieving optimal PCC and to understand the role of features based on evolutionary properties versus physicochemical properties.

First, we assessed the performance of the model when trained with all 1127 features described in the features section, labeled as ALL_MPAD. This resulted in an average PCC of 0.58 and a standard deviation (SD) of 0.09 across 50 iterations. As stated earlier, our aim was to minimize the number of features while maintaining the best performance by training the model with several subsets of features. Ultimately, this was achieved with 100 features, which include all the PSSM-associated features: normalized average PSSM scores, pseudo PSSM scores with φ = 10 for both the interacting and mutating protein sequences, and the row PSSM scores for the mutating site of the mutating sequence. This subset is labeled as Only_10_PLUS hereafter. For these optimal features, we obtained an average PCC of 0.61 and an SD of 0.08 across 50 iterations. The model obtained from the best performing iteration obtained by training with these optimal set of 100 features is ultimately implemented into the web server. To avoid overfitting during the training by 10-fold cross validation, we also performed 5-fold cross validation, which yielded similar results for both training with all the features labeled ALL_MPAD and with the optimal set of 100 features labeled ONLY_10_PLUS across 50 iterations (see Table 5). For the best performing iteration with 5-fold cross validation training, the joint plots are shown in Supplementary Fig. S4.

**Table 5.** Comparative performance of 5-fold and 10-fold cross validations (CV).

Feature Set	PCC (5-fold CV)	PCC (10-fold CV)
	50 iterations	50 iterations
ALL_MPAD	0.54 ± 0.06	0.58 ± 0.09
Only_10_PLUS	0.60 ± 0.05	0.61 ± 0.08

Next, to assess the role of features based on their category—evolutionary and physicochemical—the models were trained separately on each category. There were a total of 563 physicochemical and 460 evolutionary features. Despite the higher number of physicochemical features, the performance of the model when trained entirely on evolutionary features was significantly better than when trained on physicochemical features only. An average PCC of 0.61 (SD = 0.07) and 0.34 (SD = 0.11) across 50 iterations were obtained by training exclusively with evolutionary and physicochemical features, respectively.

**Figure 6.**
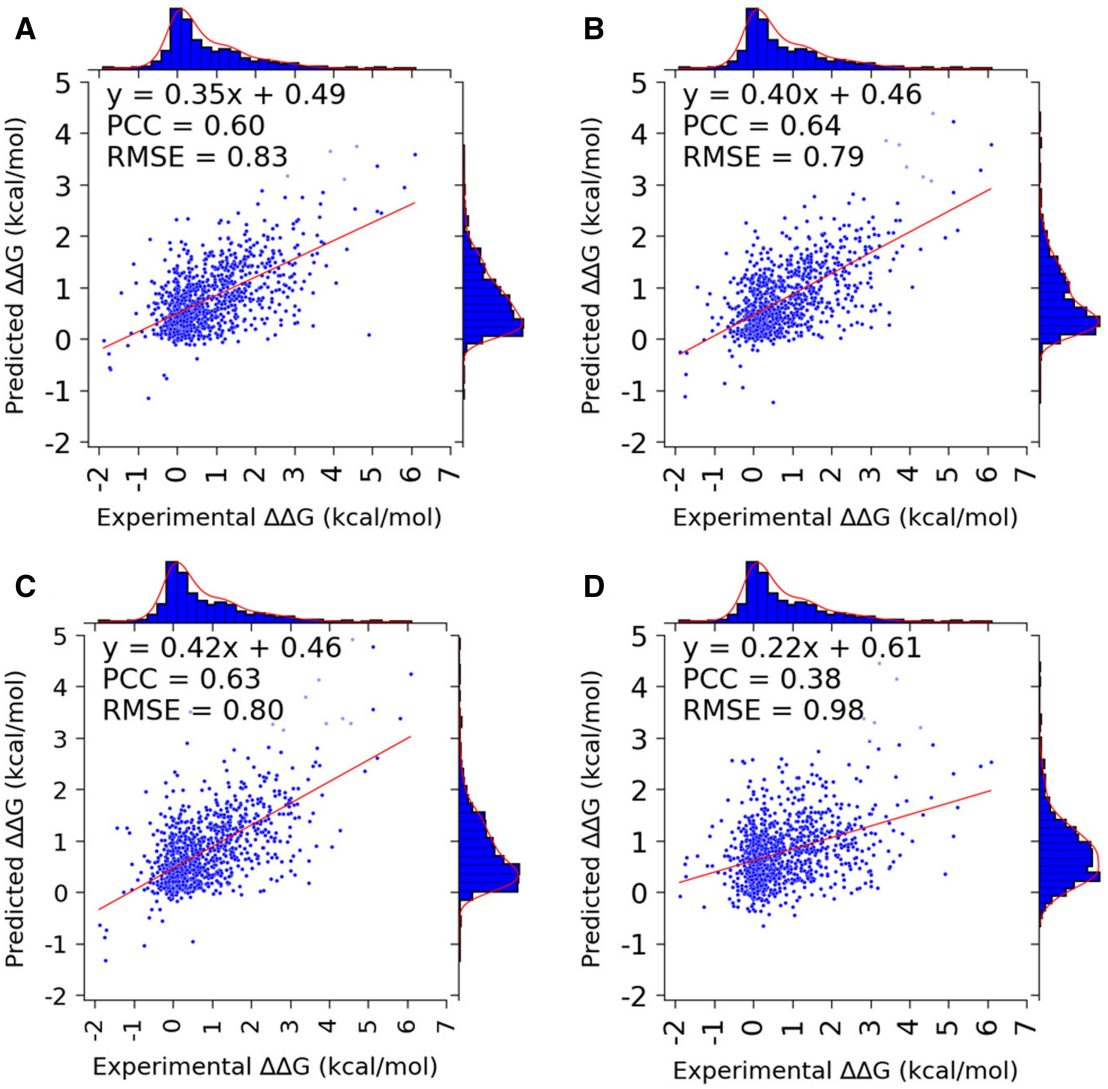
Comparison of performance of the model with (A) all the features and (B) the optimized set of features and comparison of the performance of the model with (C) evolutionary and (D) physicochemical features.

The performance of the model using all features, the optimal set of features, physicochemical features, and evolutionary features is presented in Table 6. In addition, the listing of all the features used under each of those categories is provided in Supplementary Table S3. The PCC reported in the table represents the average value across 50 iterations of training as well as for the best-performing iteration among the 50, while the plots were generated for the best-performing iteration among the 50 (Fig. 6).

**Table 6.** Comparative performance of various feature categories.

Feature description	Feature count	PCC (50 iterations)	PCC (Best)
ALL_MPAD	1127	0.58 ± 0.09	0.60
Only_10_PLUS	100	0.61 ± 0.08	0.64
Physicochemical	563	0.34 ± 0.11	0.38
Evolutionary	460	0.61 ± 0.09	0.63

### 3.4 SAAMBE-MEM web server

The final prediction model is based on the best performing iteration trained with optimal set of 100 features, namely ONLY_10_PLUS. The features used for this training are listed under Supplementary Table S3. The SAAMBE_MEM prediction model can be accessed online at the web server http://compbio.clemson.edu/SAAMBE-MEM/, where the FASTA sequences of the interacting and mutating membrane proteins are uploaded along with the mutation information, which includes the mutating site and the names of the mutating and mutated amino acids (the format can be obtained on the website). The output of the web server will be the ΔΔ*G* value predicted by the model. The prediction can also be performed locally with standalone code. In addition, the MPAD_Clean database can be downloaded from the website.

## 4 Discussion

The study presents a sequence-based approach for predicting binding free energy changes (ΔΔ*G*) due to mutations in membrane protein–protein complexes, addressing the limitations of existing methods which were trained on mostly soluble protein–protein complexes. The resulting method, the SAAMBE-MEM protocol, achieved a PCC of 0.64 utilizing only 100 features for the best performing iteration. In contrast, all tested leading ΔΔ*G* predictors, trained on the SKEMPI database, failed to deliver a PCC > 0.3. The study's methodological advancements, including rigorous feature selection and hyperparameter tuning, contribute to the robustness and reliability of the model. The availability of the prediction model via an online web server facilitates its application in various research and therapeutic contexts, enabling the broader scientific community to effectively predict ΔΔ*G* values for membrane protein complexes from sequence data.

Several observations were made throughout the investigation, namely that the distributions of ΔΔ*G* of SKEMPI and MPAD databases are not the same, MPAD ΔΔ*G* distribution strongly enriched of cases with ΔΔ*G* = 0. Combined with the observation that mutational patterns of SKEMPI and MPAD are similar, it is puzzling why MPAD has so many entries with ΔΔ*G* = 0. It suggests that interfaces of MPAD protein–protein complexes are more “plastic” than on SKEMPI allowing to rearrange, accommodate and reduce the effect of mutations. Perhaps this reflects the different nature of PPIs involving membrane and soluble proteins. Indeed, many of the entries of MPAD involve signaling and recognition of different partners (Supplementary Table S4) and thus allow for mutation tolerance.

At the same time, it was noticed that most of the entries in the MPAD database are domains or proteins that are fully or partially exposed to the water phase, and thus can be considered similar to soluble proteins. However, this contradicts the observation that the existing ΔΔ*G* predictors do not perform well (PCC < 0.3) when benchmarked on MPAD dataset. There could be numerous factors influencing the difference between MPAD and SKEMPI entries, among which one can mention the presence of the membrane. While most of the domains and proteins in MPAD are not fully embedded in the lipid bilayer, the presence of the membrane still perturbs the properties of bulk water, providing a unique environment different from soluble protein–protein complexes. Among the perturbed water properties, local pH is notably lower near negatively charged lipids compared to bulk water. In addition, there is an overabundance of various ions and an electrostatic potential originating from the membrane. While these properties were not included as features in our model since SAAMBE-MEM utilizes sequence information only, they were indirectly accounted for via the entries in the MPAD database. This resulted in the SAAMBE-MEM method, which is exclusively trained on membrane protein–protein complexes.

The binding free energy comprises two major components: enthalpy and entropy. Typically, enthalpy drives the binding through favorable interactions across the interface, while the loss of translational and rotational entropy of unbound monomers opposes the binding. However, there is a substantial difference in the entropy change for most cases in SKEMPI versus MPAD. Most entries in the MPAD database involve a partner that is already fully or partially immobilized, either bound to the membrane or connected to an integral membrane protein via a linker. Thus, the loss of entropy upon binding is much smaller for MPAD entries compared to those in the SKEMPI database. Since the distributions of wild-type binding free energy in MPAD and SKEMPI are similar but not identical (Supplementary Fig. S3) as the wild-type binding free energy of MPAD is smaller than that of the SKEMPI, the enthalpic component of MPAD cases should be smaller than that of SKEMPI. In other words, there are no strong interactions across the interface for MPAD complexes, i.e. no hot spots. Since most of the mutants in MPAD involve mutations to alanine, replacing a residue that does not have a strong interaction with the corresponding partner with alanine is expected to have little impact on the enthalpy and binding free energy change. This is why the MPAD database is enriched with cases of ΔΔ*G* close to zero.

## Supplementary Material

btae544_Supplementary_Data
