# Supplementary material for: SAAMBE-MEM: a sequence-based method for predicting binding free energy change upon mutation in membrane protein–protein complexes
Source: Bioinformatics. 2024 Sep 6;40(9):btae544. doi: 10.1093/bioinformatics/btae544 (PMC11407696; doi:10.1093/bioinformatics/btae544)
Supplement: btae544_Supplementary_Data [file btae544_supplementary_data.pdf]

SAAMBE-MEM: A Sequence-Based Method for Predicting Binding Free Energy Change upon Mutation in Membrane Protein-Protein Complexes

Prawin Rimal[1], Shailesh Kumar Panday[1], Wang Xu[2], Yunhui Peng[2], and Emil Alexov[1,*]

[1]Department of Physics and Astronomy, Clemson University, SC 29634 and [2]Institute of Biophysics and Department of Physics, Central China Normal University, Wuhan, China.

*To whom correspondence should be addressed.

Associate Editor: XXXXXXX

Received on XXXXX; revised on XXXXX; accepted on XXXXX

Table S1. Hyperparameters and their respective values used for random selection in 1000 iteration of tuning

Hyperparameters	List of Values
n_estimators	300, 400, 500, 600, 700, 800, 1000, 1200
max_depth	3, 4, 5, 6, 7, 8, 10, 12
learning_rate	0.01, 0.05, 0.08, 0.1, 0.2, 0.3, 0.5
subsample	0.1, 0.3, 0.5, 0.8, 0.9, 1.0
colsample_bytree	0.1, 0.2, 0.3, 0.5, 0.7, 0.9
reg_alpha	0.1, 0.3, 0.5, 0.8, 1.2, 1.4, 1.6, 2.0, 3.0
reg_lambda	0.1, 0.5, 1.5, 2.0, 2.5, 3.0, 3.5
gamma	0.0, 0.3, 0.5, 0.6, 0.7, 0.9

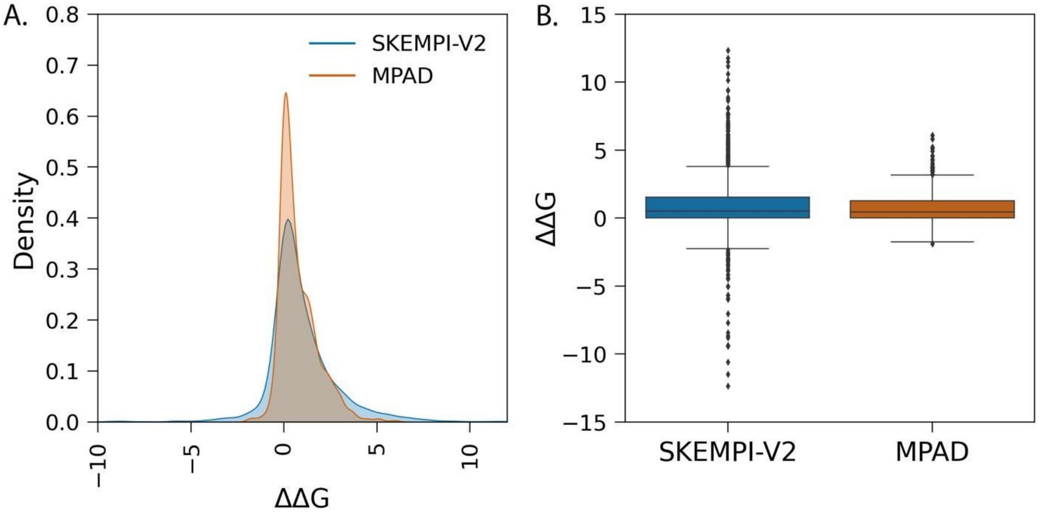

Fig S1. Comparison of SKEMPI-V2 and MPAD databases. (A) Density Plot comparing the distribution of ΔΔG values between two databases. (B) Box plots depicting the distribution of ΔΔG values.

Table S2. Number of wild-type and mutated amino acids for SKEMPI-V2 and MPAD database.

Amino Acid	SKEMPI-V2: WT	SKEMPI-V2: MT	MPAD: WT	MPAD: MT
A	120	1355	11	789
C	7	39	4	5
D	136	70	74	16
E	238	88	86	33
F	84	75	63	31
G	109	54	12	5
H	70	42	35	0
I	72	47	54	9
K	226	102	90	32
L	164	67	66	14
M	55	49	22	7
N	175	48	57	9
P	98	33	16	3
Q	83	61	53	17
R	250	80	111	18
S	127	64	79	22
T	167	45	63	7
V	78	58	39	9
W	49	51	23	7
Y	164	44	82	7
Total	2472	2472	1040	1040

Performance of Structure Based Methods for Structures Present in MPAD But Not In SKEMPI-V2

Since there is an overlap between the data present in MPAD and SKEMPI-V2, we also tested the performance of structure-based methods—SAAMBE-3D and MutaBind2—on the cases in the MPAD database with available structures of protein complexes that are not present in the SKEMPI-V2 database. This provides a gauge of the performance of the existing structure-based models when tested on cases not present in their training database. The Pearson correlation coefficients for MutaBind2[1] and SAAMBE-3D[2] were 0.25 and 0.30, respectively, indicating that there is room for significant improvement.

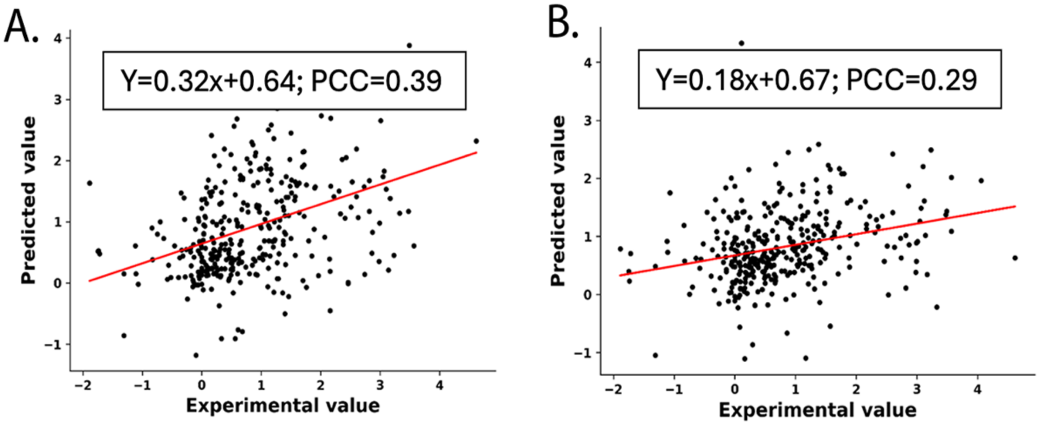

Fig S2. Performance of MutaBind2 (A) and SAAMBE-3D (B) for structures present in MPAD but not in SKEMPI-V2

Table S3. List of features used for training under various feature categories.

ALL_MPAD Features	Only_10_PLUS Features	Evolutionary Features	Physico-chemical Features
PSSM (Interacting Protein)	PSSM (Interacting Protein)	PSSM (Interacting Protein)	Amino Acid Index 1
PSSM (Mutating Protein)	PSSM (Mutating Protein)	PSSM (Mutating Protein)	Neighboring Amino Acid
Row-PSSM	Row-PSSM	Row-PSSM	Mutation Type
Pse-PSSM [φ = 1 to 10] (Interacting Protein)	Pse-PSSM [φ = 10] (Interacting Protein)	Pse-PSSM [φ = 1 to 10] (Interacting Protein)	Chemical Property
Pse-PSSM [φ = 1 to 10] (Mutating Protein)	Pse-PSSM [φ = 10] (Mutating Protein)	Pse-PSSM [φ = 1 to 10] (Mutating Protein)	Size
Amino Acid Index 1		Amino Acid Index 2	Polarity
Amino Acid Index 2			Hydrogen Bonding
Neighboring Amino Acid			Hydrophobicity
Mutation Type			
Chemical Property			
Size			
Polarity			
Hydrogen Bonding			
Hydrophobicity			
Oligomeric State			
Functional Class (Protein1)			
Functional Class (Protein 2)			
Membrane Protein Type			
pH			

Table S4. The frequency count and percentage of proteins in the MPAD database based on functional class.

Functional Class	Protein 1 Count	Protein 1 Percentage	Protein 2 Count	Protein 2 Percentage
Enzyme	73	7.02	152	14.62
GPCR	4	0.38	18	1.73
Miscellaneous	635	61.06	232	22.31
Receptor	56	5.38	401	38.56
Transporter	272	26.15	237	22.79

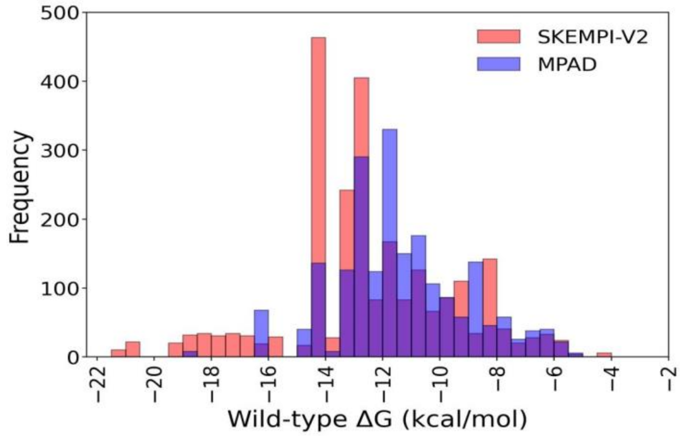

Fig S3. Distribution of wild-type ΔG for SKEMPI-V2 and MPAD Databases

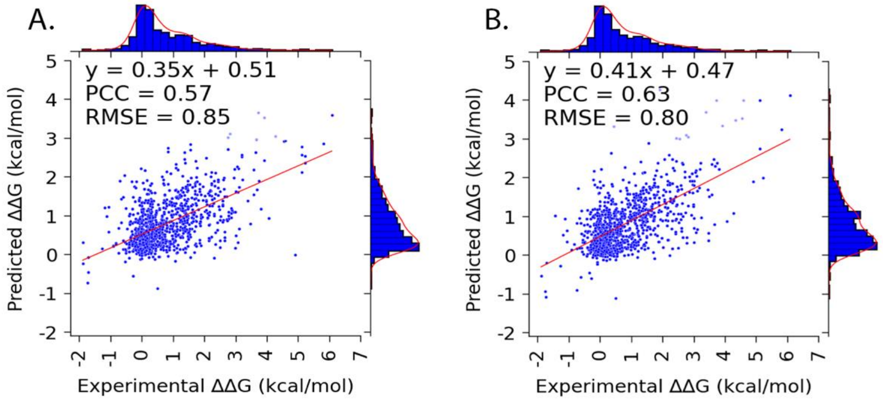

Fig S4. 5-fold cross-validation results with (A) all the features and (B) the optimized set of features

References

(1) Zhang, N.; Chen, Y.; Lu, H.; Zhao, F.; Alvarez, R. V.; Goncearenco, A.; Panchenko, A. R.; Li, M. MutaBind2: Predicting the Impacts of Single and Multiple Mutations on Protein-Protein Interactions. *iScience* **2020**, *23* (3).

(2) Pahari, S.; Li, G.; Murthy, A. K.; Liang, S.; Fragoza, R.; Yu, H.; Alexov, E. SAAMBE-3D: Predicting Effect of Mutations on Protein–Protein Interactions. *Int J Mol Sci* **2020**, *21* (7), 2563.
